# Oral Biofilms: Development, Control, and Analysis

**DOI:** 10.3390/ht7030024

**Published:** 2018-08-31

**Authors:** Daniela Berger, Aviva Rakhamimova, Andrew Pollack, Zvi Loewy

**Affiliations:** 1 Department of Pharmaceutical and Biomedical Sciences, Touro College of Pharmacy, New York, NY 10027, USA; dberger@student.touro.edu (D.B.); arakhami2@student.touro.edu (A.R.); apollack5@student.touro.edu (A.P.); 2 Department of Microbiology and Immunology, New York Medical College, Valhalla, NY 10595, USA

**Keywords:** biofilm, antimicrobial, high-throughput analysis

## Abstract

The oral cavity harbors hundreds of microbial species that are present either as planktonic cells or incorporated into biofilms. The majority of the oral microbes are commensal organisms. Those that are pathogenic microbes can result in oral infections, and at times can initiate systemic diseases. Biofilms that contain pathogens are challenging to control. Many conventional antimicrobials have proven to be ineffective. Recent advances in science and technology are providing new approaches for pathogen control and containment and methods to characterize biofilms. This perspective provides (1) a general understanding of biofilm development; (2) a description of emerging chemical and biological methods to control oral biofilms; and (3) an overview of high-throughput analytical approaches to analyze biofilms.

## 1. Introduction

Although the use of medical devices has enhanced health care and improved the quality of life, there is, unfortunately, a myriad of diseases and infections that can be attributed to biofilms associated with medical devices. Microbes can colonize on a medical device surface and cause infections, and at times can even lead to malfunction of the device. Infections are caused by a wide variety of organisms. So far, *Pseudomonas, Vibrio, Escherichia, Salmonella, Listeria, Streptococcus*, and *Mycobacteria* have been identified as causing biofilm-induced infections [1].

## 2. Biofilms

Microbes have evolved a unique survival strategy through the formation of biofilms. Many microbes evolve from the planktonic state and associate together as “communities” to form complex matrix-like structures known as biofilms [1]. Biofilms are dense micro-communities that grow on inert surfaces and encapsulate themselves with secreted polymers [2]. When organisms form a biofilm, they are able to adapt to environmental change by altering their gene expression patterns. The biofilm structure and corresponding change in gene expression can protect the microbes from disinfectant agents or antibiotics [1]. The resultant biofilm can pose a serious public health issue.

### 2.1. Biofilm Development

**Figure 1:**
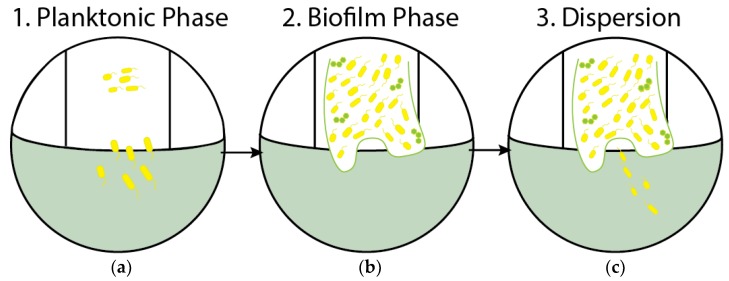
Biofilm life cycle. (**a**) Stage 1: Planktonic and free-floating bacteria make contact with a surface randomly or by chemical attraction; (**b**) Stage 2: Cells aggregate and form microcolonies on the surface; nascent biofilm is formed; (**c**) Stage 3: Dispersion phase, virulent bacteria disperse and can readily colonize other surfaces.

Biofilm development is a dynamic process [1,3]. When a planktonic microbe attaches itself to a surface, the organism can join with other microbes in the formation of a complex biofilm. Each organism has its own unique method of adhering to surfaces. Some of the modes include attachment through flagella, pili, proteins, and polysaccharide adhesins [1]. Microbes can aggregate and form biofilms on both biotic and abiotic surfaces, making it difficult to identify and isolate a therapeutic target [4]. The initial attachment of bacterial cells is the critical stage for biofilm formation. Once attachment begins, depending on the environmental conditions, the bacteria have two options. They can progress to biofilm formation by adhering to the surface, or they can revert to the planktonic phase. For those organisms that enter into a biofilm, development ceases with the onset of a dispersion phase, the stage at which virulent cells slough off from the biofilm and often cause infections in the host. Figure 1 summarizes the different stages of the biofilm life cycle.

### 2.2. Components of Biofilms

Biofilms are composed of many organisms that form a multicellular entity. The biofilm complex is held together by a matrix of excreted polymeric compounds (EPS). This matrix provides protection, adhesion, stabilization, and nutrients within the biofilm [5]. Most of the biofilm matrix (91%) is composed of water. The water can either function as a solvent or can bind directly with the microbial cells. Water is an integral component since it helps with the diffusion of the biofilm [6]. The microbial content of the biofilm is approximately 5%, the EPS matrix makes up another 2%, and DNA, RNA, and proteins another 2%. The structure of the matrix varies depending on the bacterial composition and the environmental conditions. 

### 2.3. Oral Biofilms

The oral cavity harbors diverse biofilms. Indeed, the oral cavity is unique in the level of microbial diversity, supporting up to 1000 different species of microorganisms [4]. The natural dentition and dental prostheses, including dentures and implants, are substrates for biofilms. 

#### 2.3.1. Dental Plaque

Although the oral cavity is exposed to air, the surface of the teeth may become anaerobic once colonized with bacteria [7]. Dental plaque is a poly-microbial biofilm in the mouth that can cause human infections, such as dental caries and periodontal disease [4]. Plaque contains a variety of microorganisms embedded in an extracellular matrix of polymers [8]. Plaque can also develop between the teeth and the gingival crevice making it even more difficult to remove. Infected sites are not readily treated by therapeutics; antibiotics cannot penetrate and reach the site of infection [7]. The main source of nutrients for the developing plaque within the sub-gingival crevice is gingival crevice fluid (GCF). GCF provides proteins, nutrients, and glycoproteins. Once an oral biofilm develops, proteolytic enzymes are produced that can cause direct damage to soft and hard tissues. Alternatively, they can interfere with host defense mechanisms [7].

#### 2.3.2. Dentures

Denture wearers frequently harbor biofilms that include *Candida albicans*, a pathogenic fungus that colonizes the denture material polymethyl acrylate (PMA). The prevalence of *Candida* in the mouth puts denture wearers at risk for denture stomatitis, an inflammation of the oral tissues [9]. Greater than 65% of denture wearers have stomatitis, a condition that contributes to poor oral health, poor dental hygiene, and systemic disease including diabetes [10]. 

#### 2.3.3. Implants

Dental implants are synthetic structures made of titanium and plastic materials that replace missing teeth. People with implants can develop infections and inflammation in the implant region. The bacteria associated with the implants may aggregate and form a biofilm [11]. The surface properties of the implant, including roughness, may affect bacterial adhesion to the implant and contribute to biofilm growth. The development of a pathogenic biofilm on these surfaces may result in inflammation [11]. Peri-implantitis is a destructive inflammatory process, which affects the tissue in the mouth that supports the implant.

#### 2.3.4. Biofilms, a Medical Challenge

The physical nature of biofilms, as discussed previously, and the survival mechanisms they possess, whether phenotypic adaptability or genetic resistance, leave them impervious to antibiotic treatment [12]. Given the lack of response to traditional antimicrobial therapy, biofilm infections currently pose a great challenge to the world of medicine and odontology and are responsible for many chronic infections. Some diseases/infections associated with biofilm formation on a host’s natural tissues include cystic fibrosis, native valve endocarditis, otitis media, and periodontitis. 

While different types of medical devices harbor biofilms, dental prostheses are some of the most pervasive. According to the American College of Prosthodontics (ACP), thirty-six million Americans are toothless and over a hundred-and-twenty million are missing at least one tooth. Moreover, ninety percent of people that are edentulous have dentures. With the number of edentulous people projected to increase to two-hundred million in the next fifteen years, the prevalence of infections caused by denture biofilms is certain to increase as well [13].

The presence of biofilms, especially in the elderly population who are among the most vulnerable and lack the manual dexterity to clean their dentures mechanically, can lead not only to oral infections, such as denture stomatitis, but also to fatal systemic infections, such as aspiration-pneumonia and chronic obstructive pulmonary disease (COPD) [14].

## 3. Chemical and Biological Methods to Control Oral Biofilms 

Despite the difficulty of eradicating biofilms, several conventional strategies do exist to control them. Methods to remove the biofilms include mechanical or physical methods, such as brushing. In this paper, we focus primarily on the existing chemical and biological methods. Physical removal of biofilms from dentures by brushing is common and important to help prevent infection. However, research indicates that brushing alone is not sufficient and that some form of cleanser should be added to the routine. 

### 3.1. Current Strategies

A popular approach to clean dentures uses alkaline peroxides, which come in the form of tablets. When added to water, the tablets produce an effervescent alkaline solution that generates hydrogen peroxide and active oxygen; chemicals that are able to penetrate and clean areas that brushing alone cannot reach. A study published in the Journal of Clinical Oral Investigations studied the effects of daily immersion of dentures with dental cleansers. In the study, acrylic resin dentures with a multispecies biofilm cultivated in the lab were subjected to daily, three-minute long immersions in a commercially available alkaline peroxide enzyme-containing denture cleanser. This was done for seven days and compared with a control denture group. Using colony forming units and extracellular polysaccharide formation as parameters to quantify the results, the researchers were able to demonstrate that the daily immersions decreased the total number of organisms on the dentures, including the complete eradication of *F. nucleatum* and *V. dispar* after just one day [15]. 

When comparing the effects of mechanical (i.e., brushing with dentifrice) and chemical (i.e., effervescent alkaline) denture hygiene methods, Paranhos et al. discovered that certain microbial biofilms are equally vulnerable to chemical and mechanical methods while others are not [16]. *S. aureus*, *S. mutans*, and *P. aeruginosa* were among the organisms that had the same vulnerability to either method or combination; however, *E. faecalis, C. albicans*, and *C. glabrata* displayed a greater reduction in cell numbers when either a mechanical or a combination of both methods was used [16]. 

Antiplaque oral rinses have been proven to be effective against biofilms [17]. While effective in plaque removal, rinses that contain chlorhexidine gluconate, as well as those that contain essential oils, have unwanted side effects. Tooth discoloration and occasional loss of taste are some side effects associated with the use of chlorhexidine gluconate. Essential oils do not have these unwanted side effects, and have low mammalian toxicities and are thus preferred. Various enzymes have been added to some of these mouth rinse formulations to help degrade the protein matrices of biofilms. 

Natural, herbal-based mouth rinses are also available to combat oral infections. One such product is a commercially available herbal-based, alcohol-free, film-forming, mouth rinse containing *Centella asiatica, Echinacea purpurea,* and *Sambucus nigra* [18]. Given the natural anti-inflammatory and antimicrobial properties of its components, this mouth rinse has been shown to be effective against dental plaque build-up [18]. In study participants who were asked to refrain from any other forms of oral hygiene over a two-week period, the botanical oral rinse displayed a clear advantage over control in preventing plaque formation and gingival inflammation [18]. 

Despite all currently available products, the lack of truly efficacious therapeutic options has continued to prompt the quest for novel anti-biofilm methodologies.

### 3.2. Investigational Strategies

Among investigational strategies to control biofilms, there are some that have bactericidal potential and some that have adjunctive therapy potential [19].

#### 3.2.1. Bactericidal

Bacteriophages, or bacterial viruses, can potentially be used to eradicate bacterial biofilms in a targeted and effective manner. The bacteriophage disrupts a biofilm from within the matrix by killing the bacterial cells. A study that looked at the effect of T4 phage on *E. coli* biofilms reported effective biofilm disruption [18]. Bacteriophages are easily reproducible, comparatively cheap, and have thus far not been associated with adverse events. However, they still possess problems adsorbing to bacterial target receptors and bypassing the biofilm EPS matrix. The addition of enzymes, such as polysaccharide depolymerase, may allow for penetration through the matrix. The use of bacteriophages for biofilm treatment is still investigational [19].

Antimicrobial peptides (AMPs) are another strategy to eradicate biofilm-forming bacteria [20]. AMPs can be both natural and synthetic. Natural AMPs are part of the immune system of living organisms. In humans, these short chains of amino acids that can prevent infection are expressed mainly on epithelial surfaces [20]. The mechanism of action of AMPs varies and includes disrupting bacterial cell membranes by creating hydrophilic channels, destabilizing the lipid bilayer, and even changing the curvature of the membrane. These mechanisms lead to bacterial cell penetration and death. Some natural AMPs in the oral cavity also exhibit host healing properties by inhibiting the formation of bone demineralizing cells, osteoclasts. 

To optimize specifically the antibiofilm properties of natural AMPs, synthetic AMPs have been developed. While most studies with regards to AMPs have been done on *S. mutans*, KSL, an α-helical synthetic AMP with a broad range of antibacterial activity, has been shown to be effective against thirteen different strains of bacteria. It works by destabilizing bacterial membranes through electrostatic interactions. In addition to broad-spectrum peptides, the development of narrow-spectrum synthetic peptides has been achieved through the fusion of different peptides. 

Specifically-targeted AMPs (STAMPs) allow for anti-microbial activity against specific pathogenic bacteria [20]. A study conducted by Eckert et al. assessed the antibiofilm activity of several STAMPs specific to *S. mutans* [20]. They were able to target *S. mutans* by incorporating a natural bacterial pheromone called competence-stimulating peptide (CSP) into the peptide as the targeting domain. The developed STAMP showed activity against both planktonic and biofilm *S. mutans* cells and no activity against other members of the *Staphylococcus* genus including *S. gorodonii* and *S. sanguinis* [20]. Odorless, tasteless, colorless, and no staining effects will make AMPs and STAMPs great options for oral and dental infection prevention [20]. 

#### 3.2.2. Adjunctive Therapy

Quorum sensing (QS) is a form of chemical cell-to-cell communication used by microbes to send signals in a cell-density-dependent manner [21]. First discovered in *V. fischeri*, quorum sensing systems have been identified in many other genera of bacteria, including *Pseudomonas*, *Escherichia*, and *Streptococcus*, and even fungi, such as *Candida albicans* [21,22]. Quorum sensing systems are used by microbes to control gene expression with regards to phenotype [21]. In the context of bacterial biofilms, quorum sensing is involved in key components of their life cycle, including cell dispersal, and resistance to eradication methods [21]. In *C. albicans*, QS promotes a shift in morphology to enable biofilm formation and eventually cell dispersion [22]. 

Targeting QS systems and inhibiting communication between bacterial cells offers a novel approach to combatting bacterial biofilms. Research is ongoing to find the best target within the QS system. It is important to note that while QS inhibitors can potentially hinder the maturation of biofilms, they cannot kill the planktonic bacterial cells. Therefore, Quorum Sensing Inhibitors (QSIs) can potentially be given in conjunction with antibiotics to help control biofilm infections [21].

## 4. Analytical Methods

As the importance of studying biofilm growth becomes increasingly prevalent in medical research, the need for developing new high-throughput techniques for analysis and characterization is of utmost importance. By studying biofilm growth patterns, it is believed that superior therapeutic resistance mechanisms will be elucidated. 

### 4.1. Microwell Plate Assay

One of the most common and effective methods for growing and quantifying biofilms in a high-throughput format makes use of 96-well microtiter plates. This method is reproducible, efficient, and cost-effective. The materials required for data acquisition, such as microtiter plates and plate readers, are standard equipment for most microbiology laboratories.

### 4.2. Calgary Device

The Calgary device is a variation of the 96-well format. The Calgary device is a method that allows for batch culture growth of biofilms and planktonic cells and measures the minimum inhibitory concentration (MIC), minimum bacterial concentration (MBC), and minimum biofilm eradication concentration (MBEC) of the cells. It is based on the use of a modified 96-well microtiter plate with pegs that are attached to a removable lid. The pegs are designed to be removable from the lid. This allows for microscopic observation of the biofilm structure. There are channels in the bottom chamber of the Calgary reaction vessel that allow for medium to flow across the pegs allowing for the formation of equivalent biofilms on each peg [23]. 

### 4.3. Bioflux Device

The Calgary device method is only sufficient for a static biofilm. It is also necessary to develop high-throughput methods for dynamic biofilms as these are more similar to natural biofilms than to static biofilms. The BioFlux device is a new device that is able to screen flow biofilms for viability. It uses a high-throughput system to rapidly measure fluorescence of flow biofilms with a plate reader. The BioFlux system uses microfluidics to mimic in vivo growth conditions for live cells. Each of the specially designed plates contains 96 wells. There is an input well and an output well and between them is a small channel that serves as the growth platform for cells or bacteria and allows media to flow through from the input well to the output well. An inverted microscope can be used to visualize and quantify the growth in each channel with bright field, phase, fluorescence, and confocal microscopy. The sheer flow between the wells is controlled by a system of pneumatic pumps that seal the top of the wells and pressurize them to adjust the flow [24].

### 4.4. Confocal Laser Scanning Microscopy

Confocal laser scanning microscopy (CLSM) is a very useful tool for the analysis of biofilm viability and structure. One of the biggest advantages to using CLSM is that it reduces the need to treat the biofilm before analysis, minimizing the disruption of the biofilms. CLSM has also become extremely popular due to its unique ability to provide optical thin sectioning of intact fully hydrated biofilm material, which makes it ideal for studying biofilm structure and orientation. In addition to regular imaging, CLSM is able to stack the optical sectioning images to form a three-dimensional reconstruction of the biofilm material, allowing visualization of depth and width of the biofilm. Using CLSM, biofilm cell viability can be determined using different staining methods. Cell-permeable florescent stain SYTO 59 is used to stain all cells red and the cell-impermeable florescent stain SYTOX labels only cells containing a cell wall, green. Using these two dyes together and imaging them under a CLSM using different wavelengths allows for visualization of live versus dead cells in the biofilm [25].

### 4.5. Atomic Force Microscopy

Another approach that has been used to study biofilm topography is atomic force microscopy (AFM). AFM uses an extremely sharp tip (probe) attached to a cantilever. A laser is reflected off the top of the cantilever’s flat surface and onto an optical lens that measures the movement of the cantilever and records a topographical image of the sample. There are two different ways to use AFM to measure surfaces: contact AFM and non-contact AFM. In contact AFM, the probe is dragged along the surface of the sample, and the bending of the cantilever is measured by the laser. In non-contact AFM, the probe oscillates just above the surface of the sample and as the tip approaches the surface, molecular attractions between the probe and the sample cause the oscillations to decrease. AFM has been used to characterize biofilm adhesion and binding strengths on various substrates [26]. 

## 5. Conclusions

Significant advances in our knowledge of biofilms have been achieved over the course of the past decade. The development of biofilms and their structure have been the subject of many studies. The relationship between microbial ecology and oral and systemic disease has been established. Continued efforts employing scientific and technological advancements will result in new diagnostic assays, preventative treatments, and therapeutic interventions.
